# Factors associated with the decline in under-five diarrhea mortality in India: a LiST analysis

**DOI:** 10.7189/jogh.09.020804

**Published:** 2019-12

**Authors:** Tarun Shankar Choudhary, Bireshwar Sinha, Ajay Khera, Nita Bhandari, Yue Chu, Bianca Jackson, Neff Walker, Robert E Black, Michael Merson, Maharaj Kishan Bhan

**Affiliations:** 1 Knowledge Integration and Translational Platform (KnIT) at Centre for Health Research and Development, Society for Applied Studies, New Delhi, India; 2 Ministry of Health and Family Welfare, Government of India, New Delhi, India; 3 Johns Hopkins University, Bloomberg School of Public Health, Department of International Health, Institute for International Programs, Baltimore, Maryland, United States.; 4 Duke University, Duke Global Health Institute, Durham, North Carolina, USA; 5 Indian Institute of Technology, New Delhi, India; 6 Knowledge Integration and Translational Platform (KnIT), Biotechnology Industry Research Assistance Council (BIRAC) New Delhi, India; 7 Society for Essential Health Action and Training (SEHAT), New Delhi, India

## Abstract

**Background:**

India has achieved 86% reduction in the number of under-five diarrheal deaths from 1980 to 2015. Nonetheless diarrhea is still among the leading causes of under-five deaths. The aim of this analysis was to study the contribution of factors that led to decline in diarrheal deaths in the country and the effect of scaling up of intervention packages to address the remaining diarrheal deaths.

**Methods:**

We assessed the attribution of different factors and intervention packages such as direct diarrhea case management interventions, nutritional factors and WASH interventions which contributed to diarrhea specific under-five mortality reduction (DSMR) during 1980 to 2015 using the Lives Saved Tool (LiST). The potential impact of scaling up different packages of interventions to achieve universal coverage levels by year 2030 on reducing the number of remaining diarrheal deaths were estimated.

**Results:**

The major factors associated with DSMR reduction in under-fives during 1980 to 2015, were increase in ORS use, reduction in stunting prevalence, improved sanitation, changes in age appropriate breastfeeding practices, increase in the vitamin-A supplementation and persistent diarrhea treatment. ORS use and reduction in stunting were the two key interventions, each accounting for around 32% of the lives saved during this period. Scaling up the direct diarrhea case management interventions from the current coverage levels in 2015 to achieve universal coverage levels by 2030 can save around 82 000 additional lives. If the universal targets for nutritional factors and WASH interventions can be achieved, an additional 23 675 lives can potentially be saved.

**Conclusions:**

While it is crucial to improve the coverage and equity in ORS use, an integrated approach to promote nutrition, WASH and direct diarrhea interventions is likely to yield the highest impact on reducing the remaining diarrheal deaths in under-five children.

India achieved an impressive decline in under-five mortality, from 167.5 in 1980 to 45.2 per 1000 live births in 2015 and reduced diarrhea mortality was an important contributing factor [1]. The under-five diarrheal deaths reduced by 86%; from 844 436 in 1980 to 117 285 in 2015 [2]. Despite this remarkable success, diarrhea is still the 2nd largest infectious cause of under-five mortality in India [2,3]. The Sustainable Development Goals (SDGs) now require reduction in under-five mortality to 25 or less per 1000 live births by 2030 [4,5]. An in-depth analysis of the direct and indirect factors that contributed to the decline in diarrhea deaths in the past may provide important lessons that can be applied to prevent the remaining deaths.

We report national trends in diarrhea mortality and coverage of direct diarrhea interventions and intersectoral factors known to be associated with diarrhea mortality, between 1980 and 2015. Using the Lives Saved Tool (LiST) [6-8], we assessed the attribution of these factors, individually and as packages, to the reduction in diarrhea mortality. In addition, we report projections for scaling up different intervention packages on the number of lives saved by 2030. Changes in relevant policies and programmes that may have influenced intervention coverage and under-five diarrhea mortality are also described.

## METHODS

### Data sources

The under-five and diarrhea-specific mortality data was obtained from the United Nations Inter Agency Group for Mortality Estimation (IGME) and World Health Organisation-Maternal and Child Epidemiology Estimation (MCEE) group [1,9,10]. Information on policies and programs concerning diarrhea case management, poverty reduction, health system changes, social protection schemes and scale up of intersectoral strategies to improve nutrition, access to safe drinking water and sanitation were obtained from the reports and websites of relevant ministries of the Government of India [11-19]. The intervention coverage data was sourced from the National Family Health Surveys (NFHS) and Joint Monitoring Programme reports [20-22].

### Statistical analysis

The Lives Saved Tool (*LiST*) was used to estimate the attribution of the factors, individually and as packages, in reduction of diarrhea mortality and the number of lives saved from 1980 to 2015 [6,7,23]. The *LiST* modelling approach is based on change in coverage of an intervention over a specified period, its efﬁcacy (on diarrhea specific under-five mortality), and the affected fraction (the proportion of the outcome that is amenable to treatment with the given intervention).

Coverage change × Effectiveness × Affected fraction = Impact [23]

For changes in risk factors such as stunting or wasting, their relative risk or odds ratio for cause-specific mortality are included in the model. Detailed description of the various linkages in *LiST* related to diarrhea mortality as well as the efficacy used in the model are described elsewhere [6,8]. Analysis were performed for the period 1980 to 2015 and for sub-periods, 1980-2000 and 2000-2015, representing time windows pre and post attention to achieving the Millennium Development Goals (MDG).

The year 1980 was considered as baseline for coverage data of all interventions. In case of non-availability, linear interpolation using the available estimate closest to 1980 was done to generate data, considering 1960 as the year of introduction for interventions that were available in 1980. For interventions introduced in the public sector after 1980, the year prior to introduction was considered to have zero coverage. Intervention coverage for 2015 was estimated using the most recent data source available. To obtain coverage data for the year 2000, linear interpolation was done using data of the earliest and latest measured time points. The details of the data sources are presented in the online supplementary document (Table S1 in **Online Supplementary Document**)

A predictive analysis was performed to estimate the potential impact of scaling up varied intervention packages by 2030. To guide future strategies for prevention of residual diarrhea mortality and, the potential number of lives that can be saved by upscaling different intervention packages from the coverage levels in 2015 to universal coverage levels (90%) by 2030 was explored using three scenarios. For stunting and wasting the World Health Assembly targets ie, 50% reduction in number of children stunted and wasting prevalence of <5% were used [24].

The different scenarios are as follows: Interventions in scenario 1 are related to diarrhea case management, specifically oral rehydration salt solution and zinc for all, antibiotics for dysentery and rotavirus vaccination. In scenario 2, improvement in vitamin A coverage, stunting, wasting and breastfeeding practices were additionally included and in scenario 3, Water, sanitation and hygiene (WASH) interventions ie, hand-washing with soap and combined improved sanitation and water source were added to the package in scenario 2.

## RESULTS

### Diarrhea specific mortality rate (DSMR) reduction in under-five children in India

**Figure 1:**
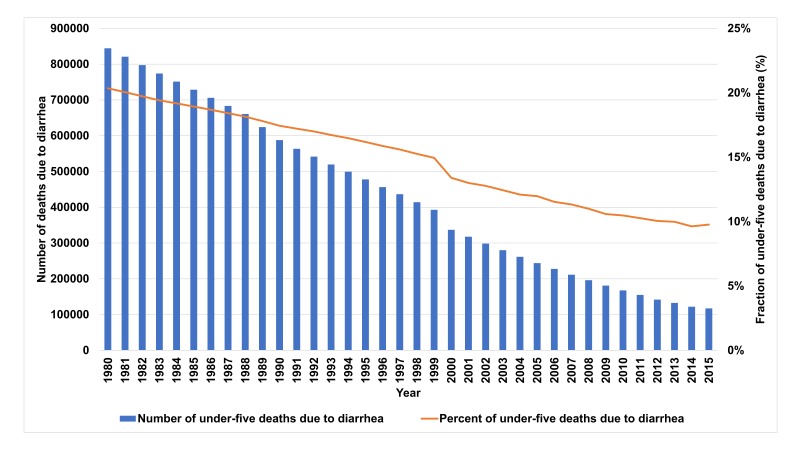
Diarrhea mortality trends in India, 1980-2015.

The under-five DSMR declined from 33.9 in 1980 to 4.7 per 1000 live births in 2015; using changes in coverage and risk factors the LiST model explains 67% of this decline [10,25]. The proportion of total under-five mortality due to diarrhea declined from 20% to 10% in the same period (**Figure 1**).

**Figure 2:**
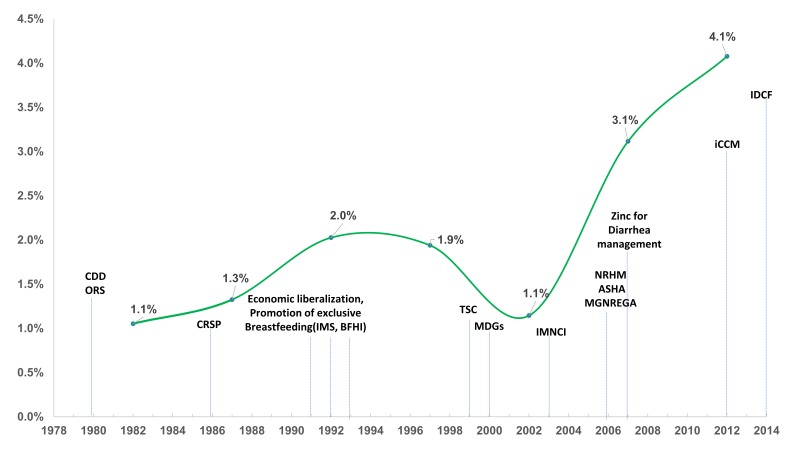
Annual rate of reduction of under-five diarrhea mortality in India, 1980-2015. CDD – Diarrhea Disease Control Programme, ORS – oral rehydration salt solution, CSRP – Central Rural Sanitation Program, IMS – infant milk substitute act, BFHI – Baby Friendly Hospital Initiative, TSC – Total Sanitation Programme, MDGs - Millennium Development Goals, IMNCI – Integrated Management of Childhood and Neonatal Illness, NRHM – National Rural Health Mission, ASHA – Accredited Social Health Activist, MGNREGA – Mahatma Gandhi National Rural Employment Guarantee Act, iCCM – integrated Community Case Management, IDCF – Intensified Diarrhea Control Fortnight [12-14,17,19,26-30].

The average annual rate of reduction in under-five diarrhea mortality per 1000 live births progressively increased from 1% to 4% between 1980 and 2014; this rate peaked post 2005 (**Figure 2**). However, the prevalence rate, based on two-week recall in national surveys, of diarrhea remained static at around 9% from 2005 to 2015 [20,21].

### Change in coverage of drivers of DSMR reduction in India

The decline in under-five diarrhea mortality coincided with the pace of economic growth in the country during the last two decades. The GDP per capita of India increased more than 5-fold from US$263 in 1980 to US$1613 in 2015 [31]; the magnitude of increase was greater in the post-2000 period. The proportion of population living below poverty line declined from 54% in 1983 to 21% in 2011 [32].

**Figure 3:**
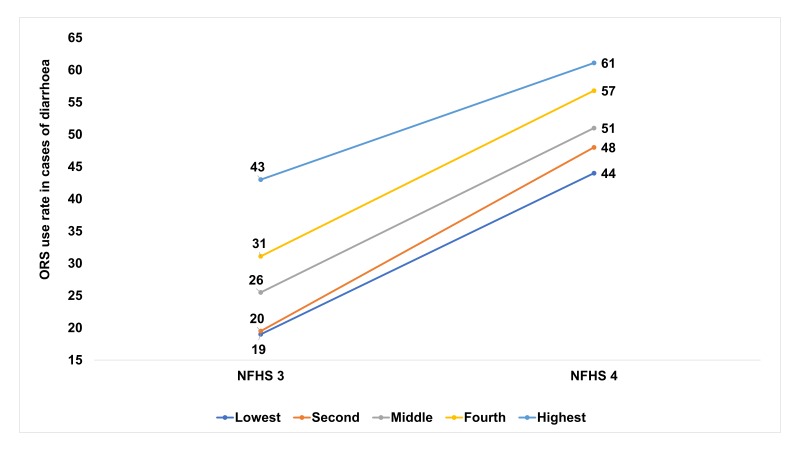
Increase in oral rehydration salt solution (ORS) use rates between NFHS-3 and NFHS-4 by wealth quintiles in India.

Data on the coverage of case management interventions, rates of wasting, stunting and access to water and sanitation are described in **Table 1**. ORS use rate during diarrhea doubled from 26% in 2005-06 (NFHS 3) to 51% in 2015-16 (NFHS 4) [20,21]. The change in ORS use rates varied by state, setting and wealth quintiles. ORS use rates were greater than 50% in three states in 2005-06 compared to 19 states in 2015-16. The ORS use in urban populations increased from 33% to 44% and that in the rural areas from 24% to 37% during this period. The increase of ORS use ranged from 18 percentage points to 29 percentage points among the different wealth quintiles and increased by 25 percentage points in the lowest wealth quintile (**Figure 3**). Zinc was introduced in 2007 for diarrhea case management [33]. Use of zinc for diarrhea treatment was 20% at national level, ranging from 13% to 58% across different states in the most recent national survey [20]. Rotavirus vaccine was introduced in the national immunization schedule in 2016 in 4 states and is being rapidly scaled to other states [34], thus could not be included in the 1980-2015 analysis but could be in the projections to 2030.

**Table 1:** Coverage data for different diarrhea related interventions and prevalence of risk factors in the year 1980, 2000 and 2015 used in the LiST analysis

Factors/Interventions	1980 (%)	2000 (%)	2015 (%)
Antibiotics for treatment of dysentery	17.1	20.5	15.8
Early initiation of breastfeeding	16.0	18.0	41.6
Hand washing with soap	10.4	15.0	17.0
Improved sanitation + improved water source	21.7	25.6	39.6
Rotavirus vaccine: two doses	0.0	0.0	0.0
ORS – oral rehydration salt solution	0.0	26.6	50.6
Persistent diarrhea treatment	0.0	0.0	33.0
Vitamin A supplementation	0.0	10.0	61.0
Zinc for treatment of diarrhea	0.0	0.0	20.3
Global stunting (<-2 SD) rate	66.2	54.3	38.4
Severe stunting rate (<1 mo)	11.9	9.5	6.8
Severe stunting rate (1-5 mo)	11.9	9.5	6.8
Severe stunting rate (6-11 mo)	17.8	14.0	10.1
Severe stunting rate (12-23 mo)	38.3	30.2	21.7
Severe stunting rate (24-59 mo)	47.9	36.2	27.2
Global wasting (<-2 SD) rate	17.3	18.1	20.9
Severe wasting (<1 mo)	10.3	11.1	13.6
Severe wasting (1-5 mo)	10.3	11.1	13.6
Severe wasting (6-11 mo)	7.6	8.4	11.0
Severe wasting (12-23 mo)	6.9	7.1	10.4
Severe wasting (24-59 mo)	3.9	4.1	7.5
Exclusive breastfeeding <1 mo	60.5	71.3	69.0
Exclusive breastfeeding 1-5 mo	41.4	44.4	44.2
Predominant breastfeeding <1 mo	18.2	16.9	18.8
Predominant breastfeeding 1-5 mo	19.0	28.2	28.4
Partial breastfeeding <1 mo	16.4	8.3	9.4
Partial breastfeeding 1-5 mo	33.9	25.8	25.9
Any breastfeeding 6-11 mo	94.9	95.1	95.0
Any breastfeeding 12-24 mo	78.1	83.9	82.4

SD – standard deviation, mo - months

There were significant improvements in nutrition related indicators in under-five children between NFHS 3 and 4. Vitamin A supplementation coverage increased from 17% to 60%, exclusive breastfeeding in first six months increased from 46% to 55%, stunting (height-for-age<-2 SD) decreased from 48% to 38%. There was a marginal increase in the proportion wasted from 20% to 21%. Households with access to an improved sanitation facility increased from 29% to 48%.

### Policies and Programs influencing DSMR reduction in India

The key policies and programs that may have influenced the rate of reduction of diarrheal mortality from 1980 to 2015 are shown in **Figure 2** and online supplementary document (Table S2 in **Online Supplementary Document**). The diarrhea control program was initiated as a vertical program in the 1980s under the effective stewardship of the Ministry of Health and Family Welfare with support from international partners including WHO and UNICEF [26]. The management of persistent diarrhea was later incorporated in the program, as it contributed to around 25% to the overall diarrhea mortality [35]. In an effort to achieve greater integration of maternal and child health interventions, diarrhea case management was incorporated as a component of the Child Survival and Safe Motherhood (CSSM) and subsequently into Reproductive and Child Health (RCH-I and RCH-II) programs [18,36,37]. Though these programs had more funding and improved management systems, integration into these broad-based programs attenuated the focus on diarrhea. Integrated Management of Neonatal and Childhood Illness (IMNCI) was introduced in 2003 for better management of common childhood illnesses and diarrhea case management became a part of IMNCI [17]. The initial scale-up and quality of practice of IMNCI was slow. The ORS supplies did not reach the periphery given the facility-based treatment focus. Subsequently, social marketing of ORS, introduction of low osmolality ORS in 2005 and zinc in 2007 were the major changes in diarrhea case management in India. The antibiotic policy for treatment of dysentery was changed to ciprofloxacin/ceftriaxone as resistance to co-trimoxazole became common [38].

The introduction of one million grass root level health workers called ASHA (Accredited Social Health Activist) was a major reform in the health system of the country under the National Health Mission in 2005 [39]. The focus then shifted to diarrhea and pneumonia treatment under integrated community case management (iCCM) [27,28]. ORS availability increased at community level through ASHA workers. Since 2014, a further boost was provided though initiation of intensified diarrhea control fortnight [29], as every family with under-five children were visited by ASHAs and delivered a packet of ORS, zinc tablets and counselling message. The diarrhea case management is now an integrated community-based program with facility linkage for management of severe cases [40].

Relevant interventions targeted at poverty reduction (The Mahatma Gandhi National Rural Employment Guarantee Scheme, 2006) and social protection for health care access (Rhastriya Swasthya Bima Yojana, 2008) were implemented across the country [11,13]. Sanitation has received major attention under Total Sanitation Campaign, earlier and now under “Swachh Bharat Mission, 2014” [12,15,16].

### *LiST* estimates for contribution of factors to DSMR reduction

**Figure 4:**
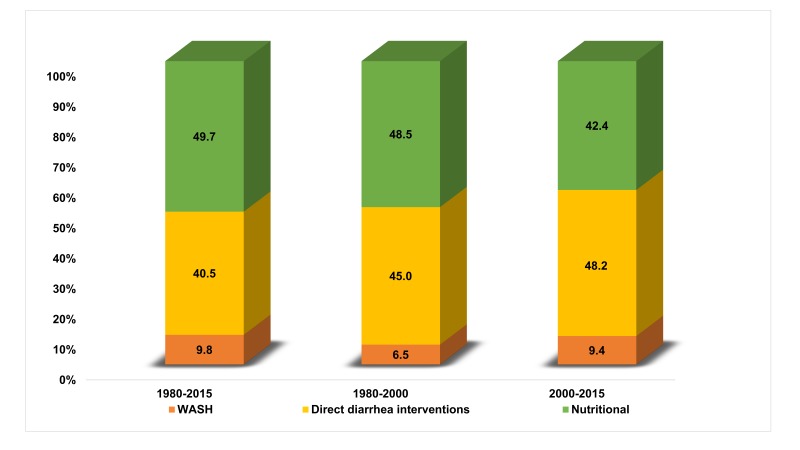
The contribution of WASH, nutritional and direct diarrhea interventions to under-five diarrhea specific mortality reduction for the time periods 1980-2015, 1980-2000 and 2000-2015.

The number of lives saved and the percentage of DSMR reduction in under-five children attributable to each factor for the periods 1980-2015, 1980-2000 and 2000-2015 are shown in **Table 2**. In comparison to 1980, over 0.66 million under-five diarrheal deaths were averted in 2015 as a combined effect of different factors. The major factors associated with DSMR reduction in under-fives were ORS use, reduction in stunting prevalence, sanitation, age appropriate breastfeeding practices, vitamin-A supplementation and treatment of persistent diarrhea. ORS use and reduction in stunting were the two key factors, each accounting for around 32% of the lives saved during 1980 to 2015. In the earlier period ie, comparing 1980 to 2000, improvements in nutrition made a higher contribution to DSMR reduction compared to diarrhea case management and WASH interventions. Whereas post-2000, diarrhea case management interventions were the major drivers for DSMR reduction (**Figure 4**).

**Table 2:** Lives saved and the percent of the diarrhea specific under-five mortality reduction attributable to each factor in India for the periods 1980-2015, 1980-2000 and 2000-2015

Factors/Intervention	1980-2015		1980-2000		2000-2015	
**Lives saved**	**Reduction attributable (%)**	**Lives Saved**	**Reduction attributable (%)**	**Lives saved**	**Reduction attributable (%)**
Zinc for treatment of diarrhea	26 198	4.0		0.0	11 462	6.6
Vitamin A supplementation	60 804	9.2	10354	3.0	21 154	12.2
ORS solution	208 701	31.6	154280	44.1	58 097	33.4
Improved water and sanitation	48 636	7.4	11136	3.2	14 494	8.3
Early initiation of breastfeeding	3204	0.5	1240	0.4	429	0.2
Changes in age-appropriate breastfeeding practices	55 637	8.4	61 863	17.7	0	0.0
Changes in wasting prevalence	0	0.0	0	0.0	0	0.0
Changes in stunting prevalence	208 907	31.6	95 968	27.4	52 091	30.0
Antibiotics for dysentery	0	0.0	3123	0.9	0	0.0
Hand washing with soap	16 077	2.4	11 740	3.4	1898	1.1
Persistent diarrhea treatment	32 590	4.9	0	0.0	14 258	8.2
Total	660 754	100.0	349 704	100.0	173 883	100.0

### LiST projections for number of lives saved attributable to scaling up different intervention packages

The projected number of lives saved and DSMR reduction in under-fives attributable to scaling up of different intervention packages for the three scenarios by 2030 are presented in **Table 3**. Our estimates show that scaling up of the direct diarrhea case management interventions (scenario 1) alone can avert around 82000 under-five diarrheal deaths in 2030; ORS use being the major contributor (51%). In addition, if the targets for nutritional parameters are also met around 20 000 additional under-five diarrheal deaths could be averted in 2030 (scenario 2). Incorporating WASH interventions to the above package will save another 3000 lives (scenario 3). Viewed another way, scaling up of the interventions packages to attain near universal (90%) coverage levels by 2030, can reduce under-five diarrhea mortality rate by 73%, 91% and 94%, for the three scenarios, respectively.

**Table 3:** Projected number of lives saved and the percent reduction in diarrhea specific under-five mortality attributable to scaling up different packages of intervention for the three different scenarios by 2030

Factors/Intervention	Direct diarrhea interventions (Scenario 1)		Direct diarrhea interventions and nutrition (Scenario 2)		Direct diarrhea interventions, nutrition and WASH (Scenario 3)	
**No. of lives saved**	**Reduction attributable (%)**	**No. of lives saved**	**Reduction attributable (%)**	**No. of lives saved**	**Reduction attributable (%)**
Zinc for treatment of diarrhea	12 820	15.7	6162	6.0	4083	3.9
Vitamin A supplementation		0.0	4848	4.7	4029	3.8
Rotavirus vaccine	9795	12.0	8358	8.2	6895	6.5
ORS solution	42 188	51.6	20 528	20.1	13 601	12.9
Improved water and sanitation		0.0		0.0	13 120	12.4
Early initiation of breastfeeding		0.0	204	0.2	182	0.2
Changes in age-appropriate breastfeeding practices		0.0	15670	15.3	12 753	12.1
Changes in wasting prevalence		0.0	16053	15.7	10 635	10.1
Changes in stunting prevalence		0.0	22191	21.7	18 332	17.4
Antibiotics for dysentery	6580	8.0	3152	3.1	2089	2.0
Hand washing with soap		0.0		0.0	16 421	15.6
Persistent diarrhea treatment	10 392	12.7	4995	4.9	3310	3.1
Total	81 775	100.0	102 161	100.0	105 450	100.0

## DISCUSSION

The highlights of this analysis are that childhood diarrhea mortality has declined substantially in India since 1980 and that this progress is related to factors that are either preventive in nature or targeted at improved case management. Preventive factors that contributed to reduce diarrheal deaths included improvements in water source, sanitation facilities, hygiene and nutrition. Improved case management resulted from use of ORS, Zinc and antibiotic treatment of dysentery. Effective case management strategies contributed substantially to diarrhea mortality reduction by preventing progression to severe diarrhea although the diarrhea prevalence has remained static. Zinc for treatment of diarrhea and rotavirus immunization were introduced recently and are yet to have much impact.

The average annual rate of reduction in diarrhea specific under-five mortality showed a 4-fold improvement coinciding with the launch of MDG-4. ORS coverage rates increased remarkably between 2005 and 2015 because of the spread of appropriate diarrhea case management from a facility to community focus. Improved logistics and availability of ASHA workers at village level improved access to ORS at households and in communities. The importance of taking care of common but potentially fatal illnesses closer to home is reflected in the substantial growth in the use of ORS among the lowest wealth quintile.

Diarrhea is still among the leading causes of under-five mortality in India. The modelling of potential impact of various intervention packages on diarrheal deaths in 2030 indicates that increased and equitable coverage of appropriate case management will have maximum impact on preventing remaining diarrheal deaths. Additional useful contributions will be through reduction in stunting, wasting and improved WASH interventions. Scaling up of rotavirus immunization will further enhance reduction in mortality and hospitalizations for severe diarrhea. A significant proportion of diarrhea mortality is related to persistent diarrhea often associated with moderate or severe malnutrition [35]. This requires increased attention as case management of diarrhea with ORS, Zinc and antibiotics for dysentery in addition to nutrition specific and sensitive interventions are scaled up. In this analysis, among under-five children, the contribution of decreased prevalence of stunting to reducing diarrhea mortality was substantial. The rates of stunting and wasting among under-fives are still high and the prevalence of wasting has not declined, which are matters of concern [20]. Malnutrition not only increases risk of mortality due to diarrhea but also pneumonia and systemic infection [41]. In this regard, the investments in improved nutrition would substantially impact overall under-five mortality and the burden of severe illness.

With effective scaling-up of simple and readily implementable measures equitably, majority of the deaths due to common illnesses such as diarrhea and others can be prevented. While what has been achieved is impressive, many diarrheal deaths that occurred could have been avoided if appropriate case management had been scaled up more effectively in the early phase of the diarrhea disease control program prior to 2005. The challenge of effective scale up and reaching the difficult-to-reach with simple affordable interventions can and must be addressed to eliminate the remaining under-five deaths.

## CONCLUSION

There has been a remarkable decline in diarrhea specific and overall under-five mortality in India since 1980. The annual rate of reduction in the diarrhea specific under-five mortality achieved a significant boost in the last decade. The progress on several fronts especially substantial reduction in poverty levels, factors directed at improving nutritional status of children, improved water and sanitation coupled with enhanced access to scalable low-cost evidence based effective case management strategies closer to home, complementing facility-based care for the more sick, are likely to have contributed to this change. The periodic redesign of the intervention delivery strategy, particularly, strengthening of home and community-based access to ORS and zinc decisively boosted coverage overall and in low socio-economic groups. Our analysis shows that while it is crucial to improve the coverage and equity in ORS use, an integrated approach to promote direct diarrhea, nutrition and WASH interventions is likely to yield the highest impact on reducing the remaining diarrhea-specific mortality in under-five children.

Unresolved challenges need to be addressed, as a large proportion of under-fives are still undernourished, without adequate sanitation and living in poor households. Innovative solutions to deliver effective case management in remote, difficult-to-reach, less penetrated areas is a priority. Improved case management packages for persistent or severe diarrhea disease also deserve attention. With rapid scale-up of rotavirus immunization in progress and these additional measures, deaths due to diarrhea should decline rapidly.

## Additional material

Online Supplementary Document
