# Supplementary material for: Factors associated with the decline in under-five diarrhea mortality in India: a LiST analysis
Source: J Glob Health. 2019 Oct 22;9(2):020804. doi: 10.7189/jogh.09.020804 (PMC6816285; doi:10.7189/jogh.09.020804)
Supplement: Online Supplementary Document [file jogh-09-020804-s001.pdf]

Online Supplementary Document

Factors associated with the decline in under-five diarrhea mortality in India: a LiST analysis

Table S1. Data sources and assumptions for India used in LiST analysis, 1980-2015.

Factor/Intervention	Data source + assumptions
Antibiotics for treatment of dysentery	1960 year of introduction and linear scale up until earliest measured data<br>NFHS 1992, NFHS 2005
Early Initiation of Breastfeeding	1980 coverage equal to earliest measured NFHS 1998, NFHS 2005, NFHS 2015
Hand washing with soap	Equal to water connection in the home in 1980 and linear scale up to earliest measured data/ 2009 global estimate
Improved sanitation + improved water source	1960 year of introduction and linear scale up until earliest measured data.<br>Joint Monitoring Programme
Rotavirus vaccine: two doses	Assumed 0 unless measured data available
ORS - oral rehydration solution	1980 year of introduction and linear scale up until earliest measured data NFHS 1998, NFHS 2005, NFHS 2015
Persistent Diarrhea Treatment	2000 year of introduction and 33% coverage worldwide in 2015
Vitamin A supplementation	0 in 1980. 1990 year of introduction and linear scale up until earliest measured data UNICEF
Zinc for treatment of diarrhea	0 in 1980 and 0 until earliest measured data NFHS 2005, NFHS 2015
Global stunting (<-2 SD) rate	Modelled stunting estimate for 1985 used for 1980 (Stevens et al, 2012) NFHS 1998, NFHS 2005, NFHS 2015
Severe stunting rate (<1 month)	
Severe stunting rate (1-5 months)	
Severe stunting rate (6-11 months)	
Severe stunting rate (12-23 months)	
Severe stunting rate (24-59 months)	
Global wasting (<-2 SD) rate	1980 prevalence equal to earliest measured NFHS 1998, NFHS 2005, NFHS 2015
Severe wasting (<1 month)	1980 prevalence equal to earliest measured NFHS 1998, NFHS 2005, NFHS 2015
Severe wasting (1-5 months)	1980 prevalence equal to earliest measured NFHS 1998, NFHS 2005, NFHS 2015
Severe wasting (6-11 months)	1980 prevalence equal to earliest measured NFHS 1998, NFHS 2005, NFHS 2015
Severe wasting (12-23 months)	1980 prevalence equal to earliest measured NFHS 1998, NFHS 2005, NFHS 2015
Severe wasting (24-59 months)	1980 prevalence equal to earliest measured NFHS 1998, NFHS 2005, NFHS 2015
Exclusive breastfeeding <1 month	1980 prevalence equal to earliest measured NFHS 1992, NFHS 1998, NFHS 2005
Exclusive breastfeeding 1-5 months	1980 prevalence equal to earliest measured NFHS 1992, NFHS 1998, NFHS 2005
Predominant breastfeeding <1 month	1980 prevalence equal to earliest measured NFHS 1992, NFHS 1998, NFHS 2005
Predominant breastfeeding 1-5 months	1980 prevalence equal to earliest measured NFHS 1992, NFHS 1998, NFHS 2005

Partial breastfeeding <1 month	1980 prevalence equal to earliest measured NFHS 1992, NFHS 1998, NFHS 2005
Partial breastfeeding 1-5 months	1980 prevalence equal to earliest measured NFHS 1992, NFHS 1998, NFHS 2005
Any breastfeeding 6-11 months	1980 prevalence equal to earliest measured NFHS 1992, NFHS 1998, NFHS 2005
Any breastfeeding 12-24 months	1980 prevalence equal to earliest measured NFHS 1992, NFHS 1998, NFHS 2005

Table S2. The timeline of various policies and programs in India which have an impact on diarrhea mortality.

Time Period	Year	Event relevant to diarrhea control
1960 to 1980	1969	Cholera Control Programme
	1970	Vitamin A prophylaxis Programme
	1975	Integrated Child Development Services
	1978	Expanded Programme on Immunization
	1980	National Diarrhea Control Programme
	1980	Introduction of Oral Rehydration Solution (ORS)
1980 to 2000	1983	National code for protection and promoting of breastfeeding
	1985	Universal Programme on immunization
	1986	Central Rural Sanitation Programme
	1992	The Infant Milk Substitute Act
	1993	Baby Friendly Hospital Initiative
	1999	Total Sanitation Campaign
	2000	Millennium Development Goals
	2000	Social marketing Oral Rehydration Solution (ORS)
2000 to 2016	2003	Integrated Management of Neonatal and Childhood Illness
	2003	The Infant Milk Substitute Act amendment
	2004	Infant and Young Child Feeding
	2005	Introduction of Accredited Social Health Activist
	2005	National Rural Health Mission
	2005	Low osmolality Oral Rehydration Solution (ORS)
	2006	Mahatma Gandhi National Rural Employment Guarantee Act
	2007	Vitamin A merged with immunization Programme
	2007	Zinc for diarrhea treatment
	2008	Rashtriya Bal Swasthya Karyakram
	2011	Home Based Newborn Care
	2011	Janani Sishu Suraksha Karyakram
	2012	Integrated Community Case Management of Pneumonia and Diarrhea
	2012	Universal Health Coverage
	2012	Child Survival Call to Action
	2013	National Health Mission
	2013	National Food Security Act
	2014	Intensified Diarrhea Control Fortnight
	2014	Mission Indradhanush
	2014	Swachh Bharat Abhiyan
	2015	The Integrated Global Action Plan for the Prevention and Control of Pneumonia and Diarrhea (GAPPD)
	2016	Rotavirus vaccine introduction
	2016	Sustainable Development Goals
	2016	Mothers’ Absolute Affection – National breast feeding Programme
